# Supplementary material for: Therapeutic effect of T-cell engager in two patients with autoimmune neuropathy
Source: Nat Commun. 2026 May 30;17:4816. doi: 10.1038/s41467-026-73819-1 (PMC13222356; doi:10.1038/s41467-026-73819-1)
Supplement: Supplementary file 1 — Supplementary Information [file 41467_2026_73819_MOESM1_ESM.pdf]

Therapeutic effect of T-cell engager in two patients with autoimmune neuropathy

Supplementary Appendix

Table of contents:

1. Supplementary Figures
2. Supplementary Tables
3. Supplementary Methods

1. Supplementary Figures

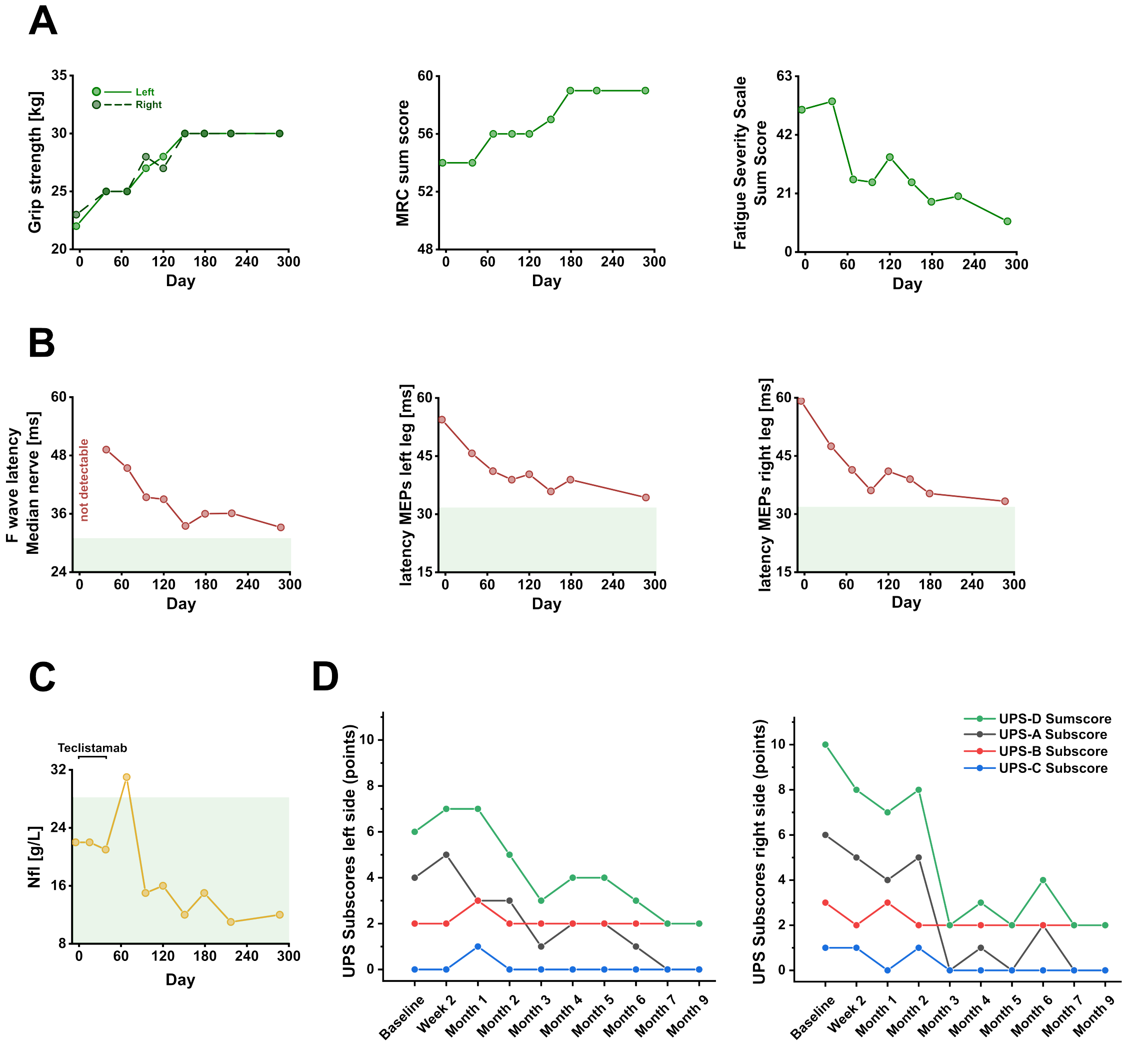

SFig. 1: Additional clinical responses and readouts to teclistamab therapy in Patient 1

A) Clinical responses: Assessment of maximal grip strength in each hand. Medical Research Council (MRC) sum score assessing muscle strength across a standard set of muscles (range 0 [most severe] to 60 [normal]) illustrating increased muscle strength after teclistamab therapy. Illustration of Fatigue Severity Scale (FSS) scores. The FSS is a 9-item self-report questionnaire designed to assess the impact and severity of fatigue in various medical conditions, ranging from 9 (least fatigue) to 63 (most severe fatigue).

B) Electrophysiological recordings: F wave latency recordings of right median nerve. Motor evoked potential (MEP) recordings of both legs. Green areas: Normal values.

C) Measurement of neurofilament (Nfl) levels showing reduction after teclistamab treatment.

D) Ultrasound pattern score (UPS) subscores that assess nerve swelling via measurement of cross-section area (CSA) in mm² at different levels. The score is divided in subscores: UPS-A comprising arm and leg sensorimotor nerves, UPS-B comprising the vagal nerve and C5 and C6 nerve roots, and UPS-C comprising sensory nerves. UPS-D is the sum of the subscores. All UPS subscores decreased after teclistamab treatment.

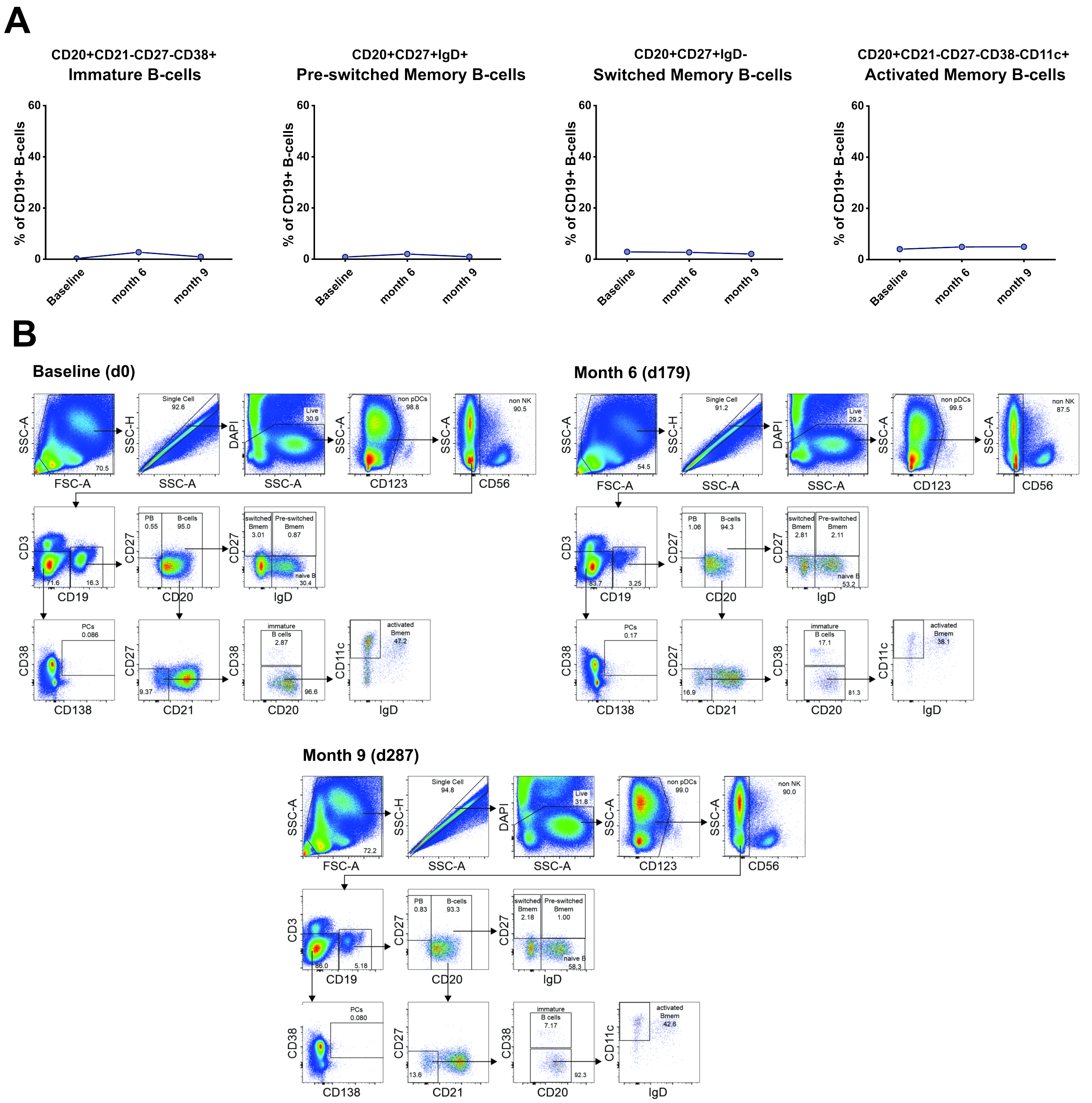

SFig. 2: Patient 1: B-cell repertoire analyzed by Fluorescence-Activated Cell Sorting in peripheral blood

A) Line graphs indicating distribution of B-cell subsets among CD19+ B-cells: Immature B-cells (CD20+CD21+CD27-CD38+), Pre-switched Memory B-cells (CD20+CD27+IgD+), Switched Memory B-cells (CD20+CD27+IgD-), and Activated Memory B-cells (CD20+CD21-CD27-CD38-CD11c+). Time points: baseline (d0, prior teclistamab) and after B-cell repopulation (d179/Month 6 and d287/Month 9).

B) Gating strategy for B-cell analysis and plasma cell analysis shown for all time points analyzed. SSC: Side Scatter, FSC: Forward Scatter, DAPI: 4′,6-diamidino-2-phenylindole; NK: Natural Killer cell; pDCs: peripheral dendritic cells; PB: Plasmablasts; PCs: Plasma cells; Bmem: Memory B-cell.

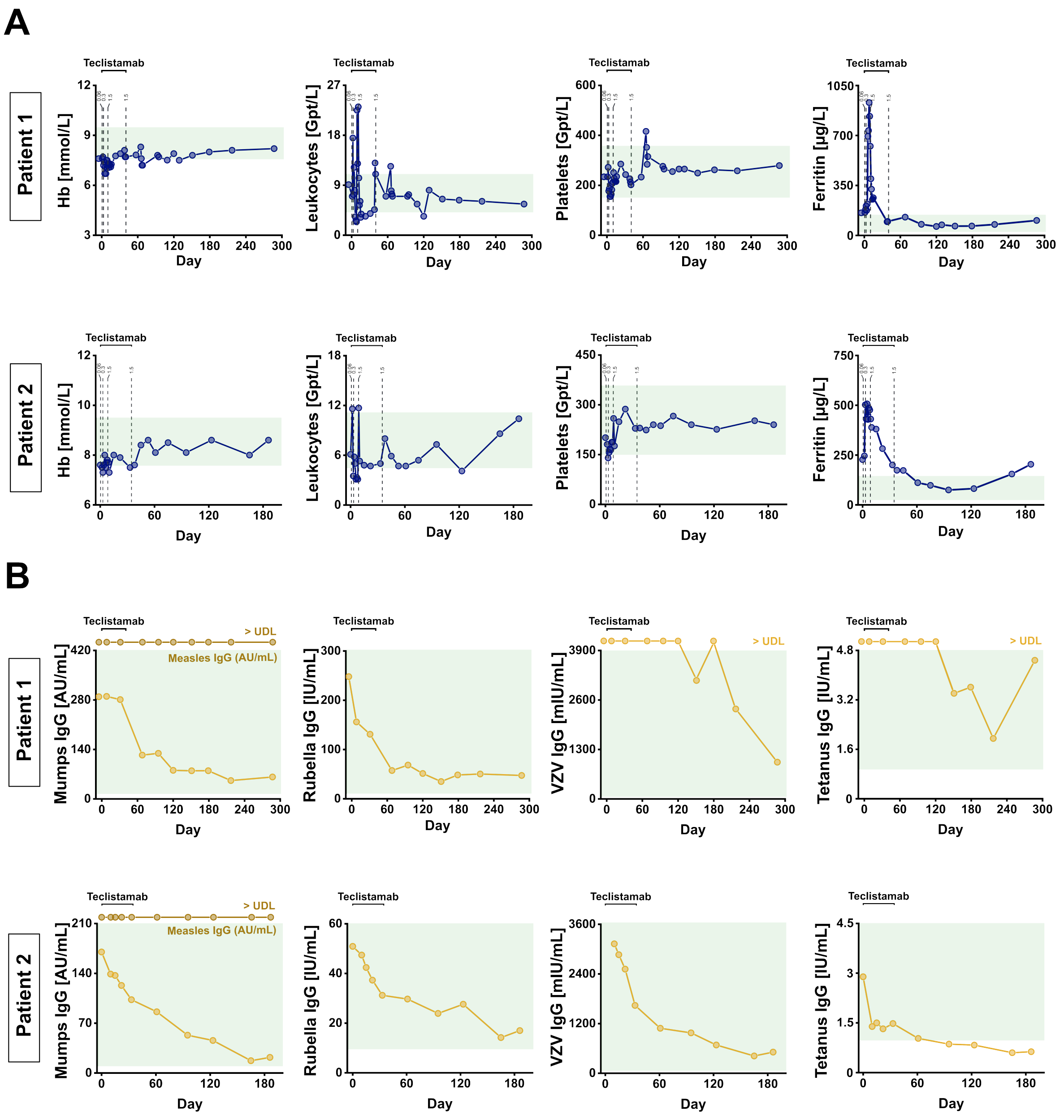

SFig. 3: Safety assessments

A) Time course of Hb, leukocyte, platelets, and ferritin levels. Dashed lines indicate time points of teclistamab injections (0.06: 0.06 mg/kg body weight, 0.3: 0.3 mg/kg body weight; 1.5: 1.5 mg/kg body weight). Green areas: Normal values.

B) Assessment of IgG antibody titers for mumps, measles, rubella, varicella-zoster virus, and tetanus. Green areas: Normal values. VZV: Varicella-Zoster Virus, UDL: Upper detection limit.

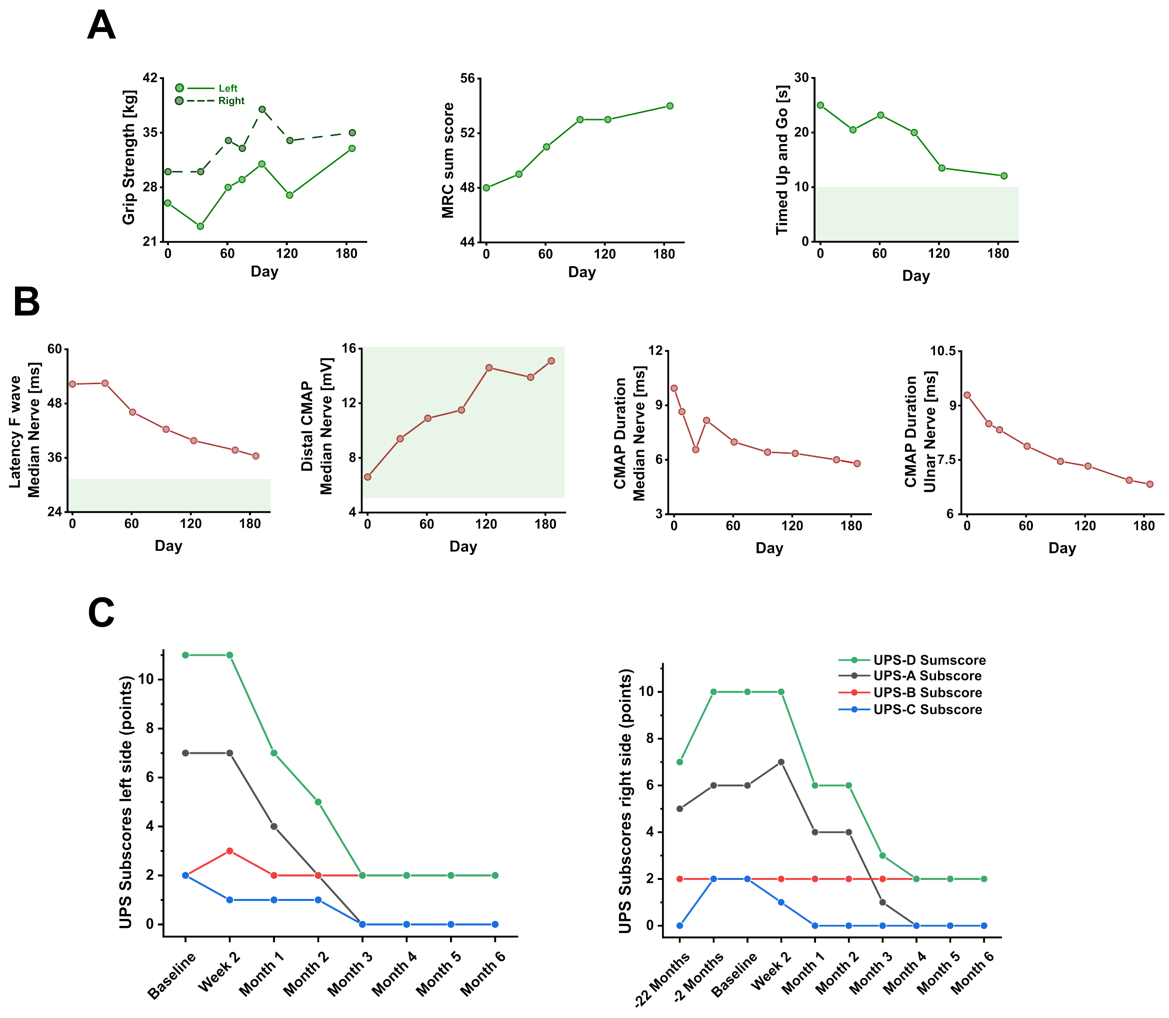

SFig. 4: Additional clinical responses and readouts to teclistamab therapy in Patient 2

A) Clinical responses: Assessment of maximal grip strength in both arms. Medical Research Council (MRC) sum score assessing muscle strength across a standard set of muscles (range 0 [most severe] to 60 [normal]) illustrating increased muscle strength after teclistamab therapy. Measurement of the Timed Up and Go test to assess functional mobility, balance, and fall risk (< 10 s: Normal mobility, green area).

B) Electrophysiological recordings: F wave latency recordings of right median and distal compound muscle action potential (CMAP) recordings of left median nerve. CMAP duration assessment of left median and right ulnar nerve. Green areas: Normal values.

C) UPS subscores and sum score of each side indicating reduced nerve swelling.

2. Supplementary Tables

Supplementary Table 1 – Preceding immunotherapy and treatment response

Patient 1

Approximate time point (before teclistamab)	Immunotherapy	Response
-2.5 years	Initial steroid pulse therapy (Methylprednisolone 1g for 5 days)	No improvement in sensory deficits; Progressive hypoesthesia and dysesthesias of both feet extending to knees and fingertips
-2.5 until -1.5 years	Intravenous immunoglobulins (IVIG), 60g every 4 weeks	Initially, improvement of sensory deficits lasting for appr. 3 weeks after IVIG therapy; after several months progressive hypoesthesia and new gait disturbance with fall and reduced maximal walking distance
-1.4 years	Steroid pulse therapy (Methylprednisolone 1g for 3 days)	No improvement of symptoms
-1.3 years	Plasma exchange	No response, slowly progressive of hypoesthesia, increase in dysasthesia; Initial paresis of the upper and lower extremities, affecting hand grip and hip flexion; further reduction in walking distance
Until teclistamab	No immunotherapy (only symptomatic treatment) as the patient temporarily declined intensification of immunotherapy	

Patient 2

Approximate time point (before teclistamab)	Immunotherapy	Response
-11.8 years	Initial steroid pulse therapy (Methylprednisolone 1g, 3 days)	Initial improvement of sensory deficits; side effect: thrombosis
-11.7 until -10.7 years	Intravenous immunoglobulins (IVIG) (30g every 4 weeks)	No effect on sensory deficits, progressive pareses
-8.8 until -8.5 years	Repetitive infusions of dexamethasone (40mg for 4 days)	Further increase of sensory deficits, progressive pareses
-8 until -7.2 years	Rituximab (200mg in two days leading to B-cell depletion)	No improvement or stabilization of symptoms; allergic reaction during first infusion
-6.5 years	Plasma exchange (5 cycles)	No response, slowly progressive sensory deficits and pareses
Until teclistamab	No immunotherapy (only symptomatic treatment) as the patient temporarily declined intensification of immunotherapy	

Supplementary Table 2 – Monthly nerve conduction studies (Patient 1)

n.p.: not performed; - : not measurable

	Nerve	Right / Left	Baseline	Month 1	Month 2	Month 3	Month 4	Month 5	Month 6	Month 7	Month 9	Normal range
**Motor nerve conduction velocity [m/s]**	Median	Right	20.1	25.1	28	28.7	30.7	33.4	33.4	31.4	41.7	> 50
	Median	Left	*n.p.*	27.5	27.4	34.3	33.8	34.7	34.2	32.6	44.2	> 50
	Tibial	Right	-	21,7	22.7	26.2	24.8	24.4	25.6	27.3	30.9	> 40.6
	Tibial	Left	-	21.4	19.7	30.0	19.1	22.2	31.1	30.2	24.2	> 40.6
	Peroneal	Right	-	18.1	-	-	24.2	24.6	24.3	26.6	28.7	> 41.7
	Peroneal	Left	14.6	17.7	19.4	21.4	22.1	24.8	24.6	27.9	27.4	> 41.7
**DML [ms]**	Median	Right	9.21	8.5	7.1	6.1	6.17	5.06	4.88	4.92	4.63	< 4.2
	Median	Left	*n.p.*	6.71	6.58	5.5	4.69	4.48	4.46	3.96	3.96	< 4.2
	Tibial	Right	8.73	8.19	10.4	8.6	6.71	4.54	3.40	4.88	4.83	< 5.1
	Tibial	Left	9.58	7.23	5.98	5.19	5.6	4.48	6.13	5.42	3.65	< 5.1
	Peroneal	Right	8.85	8.79	13.15	9.02	7.71	6.15	5.19	4.88	4.79	< 4.8
	Peroneal	Left	8.9	9.48	8.75	6.02	6.56	5.21	5.58	5.42	3.67	< 4.8
**Distal CMAP [mV]**	Median	Right	2.9	8.9	7.1	11	10.5	13.0	9.5	12.8	12.0	> 5
	Median	Left	*n.p.*	14.1	7.5	12.2	14.2	13.4	12.9	15.1	12.5	> 5
	Tibial	Right	0.1	0.5	0.5	0.9	3.0	4.4	4.8	4.0	6.4	> 5
	Tibial	Left	0.8	1.9	0.8	2.8	4.6	4.4	4.2	5.5	6.9	> 5
	Peroneal	Right	0.2	0.5	0.1	0.1	1.3	0,9	1.3	1.1	1.6	> 4
	Peroneal	Left	0.3	2.4	1.0	2.2	3.7	2.0	2.5	3.6	2.6	> 4
**F wave latency [ms]**	Median	Right	-	49.2	45.4	39.4	39.2	33.5	36.0	36.1	33.2	< 31
	Median	Left	*n.p.*	47.8	43.4	39.8	37.2	35.5	34.2	34.0	31.3	< 31
	Tibial	Right	-	-	-	-	88.5	84.6	80.9	77.4	73.1	< 63.5
	Tibial	Left	-	-	-	-	89.8	81.8	76.3	76.3	76.6	< 63.5
	Peroneal	Right	-	-	-	-	-	-	-	72.0	-	< 56.9
	Peroneal	Left	-	-	-	-	-	-	-	75.3	-	< 56.9
**Sensory nerve conduction velocity [m/s]**	Sural	Right	-	-	-	34.7	31.6	34.0	36.3	32.7	35.2	> 39.3
	Sural	Left	-	27.5	30	34.3	29.5	47.4	34.5	31.8	34.3	> 39.3
**SNAP [mV]**	Sural	Right	-	-	-	2.6	2.2	1.5	6.4	4.6	5.8	> 3.8
	Sural	Left	-	3	6.4	4.3	4.0	2.9	3.3	7.6	6.1	> 3.8

Supplementary Table 3 – Monthly nerve conduction studies (Patient 2)

n.p.: not performed; - : not measurable

	Nerve	Right / Left	Baseline	Month 1	Month 2	Month 3	Month 4	Month 5	Month 6	Normal range
**Motor nerve conduction velocity [m/s]**	Median	Right	27.1	28.3	29.7	33.9	41.3	36.7	39.4	> 50
	Median	Left	30.6	33.6	32.7	39.6	37.8	41.1	41.4	> 50
	Ulnar	Right	22.9	44.7	46.3	53.1	45.2	42.1	51.8	> 51
	Ulrnar	Left	24.9	30.6	33.6	38.6	36.9	46.0	58.5	> 51
	Peroneal	Right	-	-	-	-	-	-	-	> 41.7
	Peroneal	Left	-	-	-	-	-	-	-	> 41.7
**DML [ms]**	Median	Right	9.19	9.81	7.67	6.58	6.71	5.35	5.33	< 4.2
	Median	Left	11.5	11.19	9.02	7.96	6.27	6.56	5.58	< 4.2
	Ulnar	Right	3.54	4.58	4.71	4.38	3.83	3.27	3.42	< 3.3
	Ulrnar	Left	5.48	4.79	4.50	4.92	4.25	3.29	3.67	< 3.3
	Peroneal	Right	-	-	-	-	-	-	-	< 4.8
	Peroneal	Left	-	-	-	-	-	-	-	< 4.8
**Distal CMAP [mV]**	Median	Right	7.7	9.2	11.8	10.8	14.5	14.8	13.7	> 5
	Median	Left	6,6	9.4	10.9	11.5	14.6	13.9	15.1	> 5
	Peroneal	Right	-	-	-	-	-	-	-	> 4
	Peroneal	Left	-	-	-	-	-	-	-	> 4
**F wave latency [ms]**	Median	Right	52.3	52.5	46.1	42.3	39.8	37.7	36.4	< 31
	Median	Left	55.6	54.7	41.9	38.4	45.0	37.7	37.1	< 31
	Ulnar	Right	51.9	47.1	43.7	38.6	37.2	36.1	35.5	< 31
	Ulrnar	Left	44.8	48.4	41.8	40.4	36.4	34.6	34.8	< 31
	Peroneal	Right	-	-	-	-	-	-	-	< 56.9
	Peroneal	Left	-	-	-	-	-	-	-	< 56.9
**Sensory nerve conduction velocity [m/s]**	Sural	Right	-	-	-	-	-	-	-	> 39.3
	Sural	Left	-	-	-	-	-	-	-	> 39.3
**SNAP [mV]**	Sural	Right	-	-	-	-	-	-	-	> 3.8
	Sural	Left	-	-	-	-	-	-	-	> 3.8

3. Supplementary Methods

3.1. Treatment protocol

Previous to teclistamab administration, the vaccination status was revised. Teclistamab was administered in an inpatient setting. Before each administration of teclistamab, the patient was premedicated with 16 mg dexamethasone i.v., 50 mg diphenhydramin i.v. and acetaminophen 1 g p.o. Teclistamab was administered as follows: In patient 1: day 1 (0.06 mg/kg, i.e. 4.2 mg s.c.), day 3 (0.3 mg/kg, i.e. 21.41 mg s.c.), day 10 (initially day 5 planned [postponed due to neutropenia], 1.5 mg/kg, i.e. 106.74 mg s.c.), and day 38 (initially day 33 planned, 1.5 mg/kg, i.e. 106.74 mg s.c.). In patient 2: day 1 (0.06 mg/kg, i.e. 7.2 mg s.c.), day 3 (0.3 mg/kg, i.e. 34.3 mg s.c.), day 8 (initially day 5 planned [postponed due intermittent acustic pseudohalluzinations], 1.5 mg/kg, i.e. 180.17 mg s.c., and day 36 (initially day 33 planned, 1.5 mg/kg, i.e. 180.17 mg s.c.) Both patients were treated prophylactically with acyclovir (2x 400 mg/d) and cotrimoxazole (3x 960 mg/w). Due to cytokine release syndrome grade 2 with muscular pain, temperature up to 38.5 °C, and blood pressure above 100/80mm Hg, tocilizumab 560 mg was administered i.v. on day 5 in patient 1. No tocilizumab was needed in patient 2.

3.2. Clinical assessment

Baseline and follow-up clinical assessments were performed by the same neurologist. Disease activity was assessed by clinical neurological examination, as well as using the Rasch-built overall disability scale for immune-mediated peripheral neuropathies (I-RODS), the INCAT score, the SF-36, the painDETECT questionnaire, and the fatigue severity scale sum score. Grip strength was evaluated with a mechanical dynamometer for each hand, and the maximal walking distance (in m) was measured as the continuous distance (without stopping) on a flat track without walking aid, at normal speed.

3.3. Electrophysiological recordings

Electroneurography and motor evoked potentials (MEPs) were recorded by the same person, not involved in the treatment of the patient. Stimulus intensity was kept at the same level.

3.4. Nerve ultrasound

Nerve ultrasound was performed by a neurologist experienced in performing nerve ultrasound in patients with neuroimmunological disorders. Baseline and follow-up-recordings were performed by the same examiner.

3.5. PBMC isolation

Peripheral blood mononuclear cells (PBMCs) were isolated from whole blood using a density gradient centrifugation method. Briefly, 10–12 mL of whole blood was collected into EDTA tubes, and the blood was diluted 1:1 with phosphate-buffered saline (PBS). The diluted blood was carefully layered on top of 15 mL of Ficoll-Paque™ (Sigma Aldrich, Merck, Darmstadt, Germany, #17144002) in a 50 mL Falcon tube. The tube was centrifuged at 400g for 30 minutes at room temperature without the brake to allow for separation into distinct layers. The PBMC layer was carefully aspirated and transferred to a new 50 mL Falcon tube, avoiding contamination with the Ficoll or plasma layers. The cells were washed by adding PBS to a final volume of 50 mL, followed by centrifugation at 200g for 10 minutes at room temperature. The supernatant was discarded, and the cell pellet was resuspended in 25 mL PBS. Cell viability was assessed using trypan blue exclusion and a hemocytometer, and the cell concentration was calculated based on the average count of four large quadrants.

After a second wash step (centrifugation at 200g for 10 minutes with brake), the supernatant was discarded, and the cells were resuspended in 0.5 mL of pre-warmed CTL-C solution (ImmunoSpot #CTL-ABC, Cleveland, USA) (composed of CTL-A and CTL-B in a 4:1 ratio). The PBMCs were then gently mixed with 0.5 mL of CTL-AB solution over a 2-minute period.

The PBMC suspension was aliquoted into cryovials, with cell numbers recorded. The vials were frozen in a Mr. Frosty (Sigma Aldrich, Merck, Darmstadt, Germany, #5100-0001) freezing container for 12-48h and then stored at -80°C.

3.6. Antibody staining mix for flow cytometry

The antibody staining mix consisted of the following antibodies/reagents diluted in FACS buffer: anti-human CD8 BUV737 (1:200, clone: SK1, BD, Cat# 564629), anti-human CD19 BUV395 (1:50, clone: HIB19, BioLegend, Cat# 302298), anti-human CD123 BV711 (1:50, clone: 6H6, BioLegend, Cat# 306030), anti-human CD20 BV605 (1:100, clone: 2H7, BioLegend, Cat# 302334), anti-human CD3 BV570 (1:100, clone: UCHL1, BioLegend, Cat# 300436), anti-human CD138 BV510 (1:50, clone: MI15, BioLegend, Cat# 356518), anti-human CD14 V450 (1:50, clone: M5E2, BD, Cat# 561390), anti-human CD56 BV421 (1:100, clone: 5.1H11, BioLegend, Cat# 362552), anti-human CD38 PerCP-Cy5.5 (1:200, clone: HB-7,

BioLegend, Cat# 356614), anti-human CD45RA FITC (1:50, clone: HI100, BioLegend, Cat# 304148), anti-human CD11c PE-Cy7 (1:100, clone: 3.9, BioLegend, Cat# 301608), anti-human CD4 PE-Cy5 (1:200, clone: A161A1, BioLegend, Cat# 357430), anti-human HLA-DR PE-CF594 (1:200, clone: G46-6, BD, Cat# 562304), anti-human BCMA PE (1:50, clone: 19F2, BioLegend, Cat# 357504), anti-human CD21 APC-Cy7 (1:200, clone: Bu32, BioLegend, Cat# 354928), anti-human IgD A700 (1:50, clone: IA6-2, BioLegend, Cat# 348230), anti-human IgM APC (1:50, clone: G20-127, BD, Cat# 561010).
