# Supplementary material for: Therapeutic effect of T-cell engager in two patients with autoimmune neuropathy
Source: Nat Commun. 2026 May 30;17:4816. doi: 10.1038/s41467-026-73819-1 (PMC13222356; doi:10.1038/s41467-026-73819-1)
Supplement: Supplementary file 2 — Reporting Summary [file 41467_2026_73819_MOESM2_ESM.pdf]

nature portfolio

Corresponding author(s): Christian Geis

Last updated by author(s): Apr 13, 2026

Reporting Summary

Nature Portfolio wishes to improve the reproducibility of the work that we publish. This form provides structure for consistency and transparency in reporting. For further information on Nature Portfolio policies, see our Editorial Policies and the Editorial Policy Checklist.

Statistics

For all statistical analyses, confirm that the following items are present in the figure legend, table legend, main text, or Methods section.

n/a	Confirmed	
☐	☒	The exact sample size (*n*) for each experimental group/condition, given as a discrete number and unit of measurement
☐	☒	A statement on whether measurements were taken from distinct samples or whether the same sample was measured repeatedly
☒	☐	The statistical test(s) used AND whether they are one- or two-sided<br>*Only common tests should be described solely by name; describe more complex techniques in the Methods section.*
☒	☐	A description of all covariates tested
☒	☐	A description of any assumptions or corrections, such as tests of normality and adjustment for multiple comparisons
☒	☐	A full description of the statistical parameters including central tendency (e.g. means) or other basic estimates (e.g. regression coefficient) AND variation (e.g. standard deviation) or associated estimates of uncertainty (e.g. confidence intervals)
☒	☐	For null hypothesis testing, the test statistic (e.g. *F*, *t*, *r*) with confidence intervals, effect sizes, degrees of freedom and *P* value noted<br>*Give P values as exact values whenever suitable.*
☒	☐	For Bayesian analysis, information on the choice of priors and Markov chain Monte Carlo settings
☒	☐	For hierarchical and complex designs, identification of the appropriate level for tests and full reporting of outcomes
☒	☐	Estimates of effect sizes (e.g. Cohen's *d*, Pearson's *r*), indicating how they were calculated

Our web collection on statistics for biologists contains articles on many of the points above.

Software and code

Policy information about availability of computer code

Data collection	Natus Nicolet EDX® EMG-/ENG-/EP-/IOM-System [electroneurography]; Toshiba/Canon Aplio i800 Machine with PLT-1204BT linear array transducer (Canon Medical Systems, Otawara, Tochigi, Japan) [Nerve Sonography]; BD FACSLyric [FACS]; Magstim M2002 (Magstim Inc, Roseville, MN, USA) [motor evoked potentials]; Nfl-CLIA test (Fujirebio), BD FACSymphonyTM S6 [FACS]
Data analysis	Origin 2020b, Natus Elite® Electrodiagnostics Software [electroneurography, motor evoked potentials]; mbedded software on Toshiba/Canon Aplio i800 [nerve sonography]; BD FACSuite Clinical v1.5 [FACS], Inkscape (version 1.4.2.), FlowJo (BD Bioscience, V10), CorelDRAW 2025

For manuscripts utilizing custom algorithms or software that are central to the research but not yet described in published literature, software must be made available to editors and reviewers. We strongly encourage code deposition in a community repository (e.g. GitHub). See the Nature Portfolio guidelines for submitting code & software for further information.

Data

Policy information about availability of data

All manuscripts must include a data availability statement. This statement should provide the following information, where applicable:

- Accession codes, unique identifiers, or web links for publicly available datasets
- A description of any restrictions on data availability
- For clinical datasets or third party data, please ensure that the statement adheres to our policy

The data generated in this study are provided in the Supplementary Information and Source Data file (main figures). All numeric data of this manuscript can also be

obtained from the corresponding author upon reasonable request via email to christian.geis@med.uni-jena.de. Patient data can only be shared in pseudonymized form. Otherwise, there are no restrictions to data access.

Research involving human participants, their data, or biological material

Policy information about studies with human participants or human data. See also policy information about sex, gender (identity/presentation), and sexual orientation and race, ethnicity and racism.

Reporting on sex and gender	Sex is reported on all patients. Self-reported sex and biological sexes were identical in all patients.
Reporting on race, ethnicity, or other socially relevant groupings	There is no report on races of the patients.
Population characteristics	Age and prior disease course as well as pre-treatment is reported on all patients.
Recruitment	Patients with treatment-refractory autoimmune neuropathy were recruited at the Neurologic Department of the University Hospital in Jena between October 2024 and February 2025. Eligibility criteria were based on 1) a progressive disease course of autoimmune neuropathy mediated by a paraprotein 2) extensive pretreatment without further available on-label therapies 3) paraclinical findings: electroneurographical findings of demyelisation, pathological UPDSS scores indicating nerve swelling on ultrasound and detection of paraprotein.<br><br>There was no option for self-selection. All patients were evaluated by neurologists experienced in autoimmune neuropathies. Among 4 patients screened, two patients were selected for teclistamab therapy. The two other patients refused informed consent.<br><br>Teclistamab therapy was offered within a compassionate use setting. According to the German Arzneimittelgesetz §21/2 and the Arzneimittel-Härtefall-Verordnung §2 experimental treatment is allowed if (i) patients are afflicted by severe life-threatening disease such as progressive autoimmune neuropathy, (ii) have failed on previous treatments and (iii) in a scientific rationale exists for potential efficacy of the respective treatment in the underlying disease. The treatment was approved by the local ethics committee (Reg. No. 2024-3609-iH). All patients provided informed consent for the treatment and publication according to the CARE guidelines. No commercial sponsor was involved.
Ethics oversight	The local ethics committee of the medical faculty at the Friedrich Schiller University Jena approved the treatment of the patient under a compassionate use setting (Reg. No. 2024-3609-iH). Both patients were individually reviewed.

Note that full information on the approval of the study protocol must also be provided in the manuscript.

Field-specific reporting

Please select the one below that is the best fit for your research. If you are not sure, read the appropriate sections before making your selection.

☒ Life sciences ☐ Behavioural & social sciences ☐ Ecological, evolutionary & environmental sciences

For a reference copy of the document with all sections, see nature.com/documents/nr-reporting-summary-flat.pdf

Life sciences study design

All studies must disclose on these points even when the disclosure is negative.

Sample size	Case series of a compassionate use program. No sample size calculation is applicable. Of 4 patients screened by experienced neuroimmunologists, 2 were included. 2 did not provide informed consent.
Data exclusions	No data were excluded.
Replication	N/A. There is no replication available as this was not a study but treatment in a compassionate use setting.
Randomization	N/A. There is no control arm as this was not a study but treatment in a compassionate use setting. Treatment with a placebo in these severely affected patients would have been unethical
Blinding	N/A. There was no blinding as this was not a study but treatment in a compassionate use setting.

Behavioural & social sciences study design

All studies must disclose on these points even when the disclosure is negative.

Study description	N/A

Research sample	N/A
Sampling strategy	N/A
Data collection	N/A
Timing	N/A
Data exclusions	N/A
Non-participation	N/A
Randomization	N/A

Ecological, evolutionary & environmental sciences study design

All studies must disclose on these points even when the disclosure is negative.

Study description	N/A
Research sample	N/A
Sampling strategy	N/A
Data collection	N/A
Timing and spatial scale	N/A
Data exclusions	N/A
Reproducibility	N/A
Randomization	N/A
Blinding	N/A

Did the study involve field work? ☐ Yes ☒ No

Field work, collection and transport

Field conditions	N/A
Location	N/A
Access & import/export	N/A
Disturbance	N/A

Reporting for specific materials, systems and methods

We require information from authors about some types of materials, experimental systems and methods used in many studies. Here, indicate whether each material, system or method listed is relevant to your study. If you are not sure if a list item applies to your research, read the appropriate section before selecting a response.

Materials & experimental systems

n/a	Involved in the study	
☐	☒	Antibodies
☒	☐	Eukaryotic cell lines
☒	☐	Palaeontology and archaeology
☒	☐	Animals and other organisms
☒	☐	Clinical data
☒	☐	Dual use research of concern
☒	☐	Plants

Methods

n/a	Involved in the study	
☒	☐	ChIP-seq
☐	☒	Flow cytometry
☒	☐	MRI-based neuroimaging

Antibodies

Antibodies used

Euroimmun CA 1123-0502M (IIFT Antibodies against MAG);
Flow Cytometry:
BD Multitest™ 6-Color TBNK; Catalog No. 644611
BD Multitest™ CD3/CD8/CD45/CD4; Catalog No. 342417
BD Multitest™ CD3/CD16+CD56/CD45/CD19; Catalog No. 342416
anti-human CD8 BUV737 (clone: SK1, BD, Cat# 564629; 1:200), anti-human CD19 BUV395 (clone: HIB19, BioLegend, Cat# 302298; 1:50), anti-human CD123 BV711 (clone: 6H6, BioLegend, Cat# 306030; 1:50), anti-human CD20 BV605 (clone: 2H7, BioLegend, Cat# 302334;1:100), anti-human CD3 BV570 (clone: UCHL1, BioLegend, Cat# 300436;1:100), anti-human CD138 BV510 (clone: MI15, BioLegend, Cat# 356518;1:50), anti-human CD14 V450 (clone: M5E2, BD, Cat# 561390;1:50), anti-human CD56 BV421 (clone: 5.1H11, BioLegend, Cat# 362552;1:100), anti-human CD38 PerCP-Cy5.5 (clone: HB-7, BioLegend, Cat# 356614;1:200), anti-human CD45RA FITC (clone: HI100, BioLegend, Cat# 304148;1:50), anti-human CD11c PE-Cy7 (clone: 3.9, BioLegend, Cat# 301608;1:100), anti-human CD4 PE-Cy5 (clone: A161A1, BioLegend, Cat# 357430;1:200), anti-human HLA-DR PE-CF594 (clone: G46-6, BD, Cat# 562304;1:200), anti-human BCMA PE (clone: 19F2, BioLegend, Cat# 357504;1:50), anti-human CD21 APC-Cy7 (clone: Bu32, BioLegend, Cat# 354928;1:200), anti-human IgD A700 (clone: IA6-2, BioLegend, Cat# 348230;1:50), anti-human IgM APC (clone: G20-127, BD, Cat# 561010;1:50).

Validation

All antibodies are validated for their respective target on human cells as provided by the information provided by the manufacturer. Validation reports are available under: https://www.euroimmun.com; https://www.bdbiosciences.com/en-eu by entering the respective catalogue number

Eukaryotic cell lines

Policy information about cell lines and Sex and Gender in Research

Cell line source(s) — N/A

Authentication — N/A

Mycoplasma contamination — N/A

Commonly misidentified lines (See ICLAC register) — N/A

Palaeontology and Archaeology

Specimen provenance — N/A

Specimen deposition — N/A

Dating methods — N/A

☐ Tick this box to confirm that the raw and calibrated dates are available in the paper or in Supplementary Information.

Ethics oversight — *Identify the organization(s) that approved or provided guidance on the study protocol, OR state that no ethical approval or guidance was required and explain why not.*

Note that full information on the approval of the study protocol must also be provided in the manuscript.

Animals and other research organisms

Policy information about studies involving animals; ARRIVE guidelines recommended for reporting animal research, and Sex and Gender in Research

Laboratory animals	N/A
Wild animals	N/A
Reporting on sex	N/A
Field-collected samples	N/A
Ethics oversight	N/A

Note that full information on the approval of the study protocol must also be provided in the manuscript.

Clinical data

Policy information about clinical studies

All manuscripts should comply with the ICMJE guidelines for publication of clinical research and a completed CONSORT checklist must be included with all submissions.

Clinical trial registration	N/A There was no clinical trial registration as this was not a study but treatment in a compassionate use setting. Please see comments on ethics and recruitment mentioned above (Research involving human participants)
Study protocol	N/A There was no study protocol as this was not a study but treatment in a compassionate use setting.
Data collection	Prospective data collection was performed from January 2025 until August 2025 at Jena University Hospital, Germany
Outcomes	Assessments and outcomes were pre-specified and included baseline and follow-up clinical parameters, laboratory analyses, electrophysiological studies, and nerve ultrasound

Dual use research of concern

Policy information about dual use research of concern

Hazards

Could the accidental, deliberate or reckless misuse of agents or technologies generated in the work, or the application of information presented in the manuscript, pose a threat to:

No	Yes	
☒	☐	Public health
☒	☐	National security
☒	☐	Crops and/or livestock
☒	☐	Ecosystems
☒	☐	Any other significant area

Experiments of concern

Does the work involve any of these experiments of concern:

No	Yes	
☒	☐	Demonstrate how to render a vaccine ineffective
☒	☐	Confer resistance to therapeutically useful antibiotics or antiviral agents
☒	☐	Enhance the virulence of a pathogen or render a nonpathogen virulent
☒	☐	Increase transmissibility of a pathogen
☒	☐	Alter the host range of a pathogen
☒	☐	Enable evasion of diagnostic/detection modalities
☒	☐	Enable the weaponization of a biological agent or toxin
☒	☐	Any other potentially harmful combination of experiments and agents

Plants

Seed stocks	N/A
Novel plant genotypes	N/A
Authentication	N/A

ChIP-seq

Data deposition

☐ Confirm that both raw and final processed data have been deposited in a public database such as GEO.

☐ Confirm that you have deposited or provided access to graph files (e.g. BED files) for the called peaks.

Data access links<br>*May remain private before publication.*	N/A
Files in database submission	N/A
Genome browser session<br>(e.g. UCSC)	N/A

Methodology

Replicates	N/A
Sequencing depth	N/A
Antibodies	N/A
Peak calling parameters	N/A
Data quality	N/A
Software	N/A

Flow Cytometry

Plots

Confirm that:

☒ The axis labels state the marker and fluorochrome used (e.g. CD4-FITC).

☒ The axis scales are clearly visible. Include numbers along axes only for bottom left plot of group (a 'group' is an analysis of identical markers).

☒ All plots are contour plots with outliers or pseudocolor plots.

☒ A numerical value for number of cells or percentage (with statistics) is provided.

Methodology

Sample preparation	Peripheral blood mononuclear cells (PBMCs) were isolated from whole blood using density gradient centrifugation with Ficoll-Paque (Sigma Aldrich, Merck, Darmstadt, Germany, #17144002), followed by careful aspiration of the PBMC layer. After washing and assessing cell viability, the PBMCs were resuspended in a CTL-C solution and mixed with CTL-AB solution (ImmunoSpot #CTL-ABC, Cleveland, USA). The cells were aliquoted into cryovials, frozen in a Mr. Frosty container (Sigma Aldrich, Merck, Darmstadt, Germany, #5100-0001) , and stored at -80°C for future use. For flow cytometry, PBMCs were thawed, diluted in FACS buffer, and centrifuged before being filtered and stained with an antibody mix on ice. The cells were washed multiple times, resuspended in DAPI-containing FACS buffer, and filtered through a 30 µm cell strainer. Finally, the samples were acquired using a BD FACSymphony S6 flow cytometer, and data analysis was performed using FlowJo software.For CD19+ B- and T-cell quantification, sample preparation was performed according to the instructions of the test kit manufacturer (BD Multitest 6-Color TBNK, Cat. No. 644611).

Instrument	Samples were measured using a BD FACSLyric and BD FACSymphonyTM S6
Software	Flow cytometry data were analyzed using BD FACSuite Clinical v1.5 and FlowJo (BD Bioscience, V10)
Cell population abundance	0 - 75.28%
Gating strategy	Gating strategy for the identification of the B cell subsets. After exclusion of doublets, debrits, dead cells, CD123+pDCs and CD56+ NK cells, different B cell subets were identified within CD3-CD19+ cells or within CD3-CD19- cell population (essentially CD38+CD138+ plasma cells). The gating strategy is shown in SFig 2B. Sequential gating was applied to identify CD3+ T-cells and CD19+ B-cells populations, starting from live, singlet, CD45+ lymphocytes.

☒ Tick this box to confirm that a figure exemplifying the gating strategy is provided in the Supplementary Information.

Magnetic resonance imaging

Experimental design

Design type	N/A
Design specifications	N/A
Behavioral performance measures	N/A

Acquisition

Imaging type(s)	N/A
Field strength	N/A
Sequence & imaging parameters	N/A
Area of acquisition	N/A

Diffusion MRI ☐ Used ☒ Not used

Preprocessing

Preprocessing software	N/A
Normalization	N/A
Normalization template	N/A
Noise and artifact removal	N/A
Volume censoring	N/A

Statistical modeling & inference

Model type and settings	N/A
Effect(s) tested	N/A

Specify type of analysis: ☐ Whole brain ☐ ROI-based ☐ Both

Statistic type for inference (See Eklund et al. 2016)	*Specify voxel-wise or cluster-wise and report all relevant parameters for cluster-wise methods.*
Correction	*Describe the type of correction and how it is obtained for multiple comparisons (e.g. FWE, FDR, permutation or Monte Carlo).*

Models & analysis

n/a	Involved in the study
☒	☐ Functional and/or effective connectivity
☒	☐ Graph analysis
☒	☐ Multivariate modeling or predictive analysis

Functional and/or effective connectivity	N/A
Graph analysis	N/A
Multivariate modeling and predictive analysis	N/A
